# Quality assessment of clinical practice guidelines on psychological distress of cancer patients using the AGREE II instrument

**DOI:** 10.3389/fonc.2022.942219

**Published:** 2022-08-09

**Authors:** Ran Hao, Haoyu Jin, Jinfan Zuo, Rumeng Zhao, Jie Hu, Yixin Qi

**Affiliations:** ^1^ Department of Clinical Humanistic Care and Nursing Research Center, School of Nursing, Hebei Medical University, Shijiazhuang, China; ^2^ Department of Science and Technology, Hebei Medical University, Shijiazhuang, China; ^3^ Department of Breast Center, The Fourth Hospital of Hebei Medical University, Shijiazhuang, China

**Keywords:** cancer, psychological distress, clinical practice guideline, AGREE II, quality assessment

## Abstract

**Objective:**

This study aimed to assess the quality of the clinical practice guidelines on psychological distress among cancer patients and provide users with recommendations for coping with psychological distress.

**Methods:**

A systematic search of relevant clinical practice guidelines was undertaken to identify and select the clinical practice guidelines related to psychological distress among cancer patients. Literature databases were searched in PubMed, Web of Science, Excerpta Medica Database, the Cumulative Index to Nursing & Allied Health Literature, China Biology Medicine, China National Knowledge Infrastructure, WanFang and Weipu Journal Database. The guideline databases include Yimaitong Guidelines Network, National Guideline Clearinghouse, National Institute for Health and Clinical Excellence, American Society of Clinical Oncology (ASCO), New Zealand Guidelines Group, Scottish Intercollegiate GuidelinesNetwork, American Psychological Association, Registered Nurses’ Association of Ontario and Cancer Care Ontario (CCO). Four independent reviewers assessed the eligible guidelines using the Appraisal of Guidelines for Research and Evaluation (AGREE II) instrument.

**Results:**

Six clinical practice guidelines were included and assessed for critical evaluation. The median score for the scope and purpose domain was 71.5% (IQR 64%-77.25%), the stakeholder involvement domain was 65% (IQR 47.5%-74.5%), the rigour of the development domain was 61.5% (IQR 45.5%-85.25%), the clarity of the presentation domain was 91% (IQR 72.25%-94.5%), the applicability domain was 70% (IQR 33%-78.75%), and the editorial independence domain was 48.84% (IQR 61.75%-95%). Four guidelines (ASCO, 2014; Canadian Association of Psychosocial Oncology, 2015; NCCN, 2020, and CCO, 2016) were classified as “recommended,” and the remaining (European Palliative Care Research Collaborative and Chinese Psychosocial Oncology Society) were “recommended with modifications,” especially in the domains of Stakeholder involvement, rigour of development, and applicability. The inter-rater consistency of each domain showed moderate level (0.52–0.90) analyzing by intraclass correlation.

**Conclusions:**

The clinical practice guidelines on psychological distress among cancer patients varied in quality, and there were discrepancies in terms of the recommendations and recommendation grades. These findings could contribute to improving the quality of clinical practice guidelines on psychological distress, and enable the development and implementation of evidence-based guidelines for cancer patients.

**Systematic Review Registration:**

https://www.crd.york.ac.uk/PROSPERO, identifier CRD42020209204.

## Introduction

Cancer has become a major public health concern that poses a serious threat to human health (1). In 2022, there is an estimated 1,918,030 new cancer cases and 609,360 mortalities from cancer in the United States (2). Besides, it is expected that there will be approximately 4,820,000 people newly-diagnosed with cancer, and 3,210,000 people dying from cancer in China (3). Cancer patients often experience psychological distress, a multifactorial and unpleasant experience that includes psychological (e.g., cognitive, behavioral, emotional), social, spiritual and/or somatic states that may affect a patient’s ability to cope with their cancer, somatic symptoms, and treatment (4). Psychological distress can affect the progression and prognosis of cancer. Batty et al. (5) showed that cancer patients with psychological distress had a higher mortality rate, and apart from physical factors, psychological distress might be another predictor of cancer death.

Presently, assessment and management of psychological distress among cancer patients remain the major challenge for clinicians and vary significantly in clinical practice (6). Ideally, evidence-based guidelines have been systematically developed to combine current evidence that will aid physician clinical decision-making for specific clinical circumstances (7). The usefulness of guidelines primarily depends on the quality, rigorous methodology, and transparency of development (8). However, systematic evaluation of existing guidelines related to psychological distress of cancer patients is still lacking. In view of this, our aim was to critically appraise existing guidelines of psychological distress among cancer patients.

Besides, according to systematic reviews of oncology-related guidelines in recent years (9–13), guidelines have not always been developed in accordance with the generalized specifications. Some guidelines do not provide sufficient information to facilitate their application in real circumstances (9–11). Gao et al. (14) evaluated 98 clinical practice guidelines (CPGs) for diabetes mellitus published in China, and found 84 out of 98 CPGs rarely provided facilitators and barriers of its application, or the potential resource implications of applying the recommendations, which resulted in imperfect application of the CPGs in clinical practice. If the guidelines are not developed in strict adherence to the generalized specifications, it could result in a lack of methodological rigour in guideline development (12). Alternatively, some guidelines do not provide the potential detailed conflict of interest statement, leading to a low level of editorial independence (13). So, the quality of the guidelines developed by diverse organizations varies widely. Using recommendations that are proposed based on poor-quality guidelines may produce negative impacts on patient outcomes (15).

The Appraisal of Guidelines for Research and Evaluation II (AGREE II) is a reliable and useful tool for the assessment of guidelines (16–18). In fact, in a systematic review comparing 24 different appraisal tools for clinical guidelines, AGREE II was shown to be the most effective (19). It consists of 23 items that belong to six domains (scope and purpose, stakeholder involvement, rigor of development, clarity of presentation, applicability, and editorial independence). Previous studies have used AGREE II for the quality assessment of clinical practice guidelines for cancer cachexia, newborn hearing screening, breast cancer treatment, eosinophilic esophagitis, oral cancer treatments, and so on (19–23). In this study, we applicated the AGREE II instrument to appraise the quality of the existing guidelines for psychological distress among cancer patients.

Therefore, through systematic searching the literature and guideline databases, we would thoroughly review and appraise the guidelines on the management of psychological distress among cancer patients. We sought to assess their methodological quality by using the AGREE II instrument, to dentify the gaps limiting evidence based practice, and to provide the appropriate recommendation for healthcare workers to alleviate the psychological distress among cancer patients.

## Methods

### Study design

This study conducted a systematic review of clinical guidelines related to psychological distress among cancer patients using the AGREE II instrument.

### Review protocol

This study was performed following the guidelines from the preferred reporting items for systematic reviews and meta-analyses (PRISMA) (24). The study was registered in the International Prospective Register of Systematic Reviews (PROSPERO), and the approved number was CRD42020209204.

### Searches strategy

We searched the CPGs in literature databases and guidelines databases from January 2011 to January 2020. The literature databases included PubMed, Web of Science, Excerpta Medica Database (EMBASE), the Cumulative Index to Nursing & Allied Health Literature (CINAHL), China Biology Medicine (CBM) disc, China National Knowledge Infrastructure (CNKI), WanFang database and Weipu Journal Database. The search keywords included “Neoplasm*,” “Cancer*,” “consensus,” “guidance,” “Guideline,” “Anxiet*,” “Depressi*,” and “distress”. The searches strategy was listed in **Tables S1**, **S2**. We have also searched the guideline databases for the latest version of guidelines: YiMaiTong database, National Guideline Clearinghouse (NGC), National Institute for Health and Clinical Excellence (NICE), American Society of Clinical Oncology (ASCO), New Zealand Guidelines Group (NZGG), Scottish Intercollegiate Guidelines Network (SIGN), American Psychological Association (APA), Registered Nurses’ Association of Ontario (RNAO) and Cancer Care Ontario (CCO). The websites of all the databases were listed in **Table S3**.

### Study selection and data extraction

In this study, we performed a systematic search of clinical practice guidelines of psychological distress in cancer patients from literature databases and guideline databases. Then, the duplicate records were removed using EndNote X8 literature management software. Two reviewers independently screened the guidelines by reviewing the titles, abstracts, and full-text to identify eligible guidelines. The inclusion criteria were as follows: (i) complete guideline text is available in English or Chinese; (ii) guideline contains recommendations regarding the management of cancer patients’ psychological distress; (iii) the guidelines were published in the last 10 years. If the guideline had more than 1 version, only the most up-to-date version was assessed. The exclusion criteria were as follows: (i) duplicate guidelines; (ii) editorials and short summaries; and (iii) the interpretations or translations of guidelines and the appraisal of guideline application.

Two reviewers independently extracted the relevant information from each eligible guideline. Disagreements were resolved by consensus or the third expert. The following characteristics of the guidelines were collected: guideline’s title, the organization that created the guideline, year of publication, the publication country, guideline’s topic, and guideline version (**Table 1**).

**Table 1:** Characteristics of included guidelines.

Guideline ID	Development organization	Version	Grading system	Nation	Topic
ASCO, 2014 (25)	American Society of Clinical Oncology	Original version	–	America	Depression, anxiety
EPCRC, 2011 (26)	European Palliative Care Research Collaborative	Original version	GRADE	EU	Depression
CAPO, 2015 (27)	Canadian Association of Psychosocial Oncology	Update	GRADE	Canada	Psychological pain, depression, anxiety
NCCN, 2020 (28)	National Comprehensive Cancer Network	Update	NCCN	America	Psychological pain
CPOS, 2020 (29)	Chinese Psycho-social Oncology Society	Update	GRADE	China	Psycho-Oncology
CCO, 2016 (30)	Cancer Care Ontario	Update	CNMAT	Canada	Depression

ASCO, American Society of Clinical Oncology; EPCRC, European Palliative Care Research Collaborative; EU, European Union; CAPO, Canadian Association of Psychosocial Oncology; NCCN, National Comprehensive Cancer Network; CPOS, Chinese Psycho-social Oncology Society; CCO, Cancer Care Ontario.

### Quality assessment of guidelines

Four experts was invited to conduct the quality assessment of CPGs, and their research fields included psychology, psychiatry, clinical oncology, and oncology nursing (**Table 2**). The experts, all with professional title of associate senior or above, had 11 to 30 years of experience in their field, were familiar with this study. They all possessed the capacity of evaluating the guidelines related to the management of psychological distress among oncology patients.

**Table 2:** Experts information for evaluating of included guidelines.

Expert	Age	Gender	Educational Background	Clarity and presentation	Research Orientation	Work Place	Work Year
N1	55	female	Doctor	Professor	Mental health, Oncology care	University	30
N2	41	female	Master	Associate Chief Physician	Journal of Oncology	Department of Radiotherapy	13
N3	38	female	Doctor	Associate Chief Physician	Mental Health	Department of Psychiatry	11
N4	48	female	Doctor	Professor	Mental health care	University	23

According to the AGREE II manual, each guideline that met our inclusion criteria was scored on 23 items within six domains. Domain 1 (scope and purpose) included three items: guideline objectives, health questions, and population application. Domain 2 (stakeholder involvement) was based on three items: guideline development group, preference of target population, and target users. Domain 3 (rigour of development) included eight items: systematic methods used to search evidence, criteria for selection, strengths, and limitations of the evidence, methods for formulating the evidence, health benefits and side effects of recommendations, explicit links between recommendation and supporting evidence, expert reviewers, and updating guidelines for future use. Domain 4 (clarity and presentation) was divided into three items: recommendations that are specific and unambiguous, different options for management, and key recommendations. Domain 5 (applicability) consisted of four items: facilitators and barriers, advice/tools to implement recommendations into practice, resources for implications, and auditing criteria. Domain 6 (editorial independence) was divided into two items: editorial independence from the funding body and conflicts of interest of the guideline development members.

Based on the domain scores of each guideline, we identified them into three grades: recommended, recommended with modifications, and not recommended (31, 32). The number of domain scoring > 60% determined the grade of each guideline. The guideline was defined as “recommended” with five or six domains scoring > 60%, “recommended with modification” with three or four domains scoring > 60%, and “not recommended” with two or fewer domains scoring > 60%.

### Data analysis

A 7-point scale was used to score each item ranging from 1 strongly disagree, to 7 strongly agree. The domain scores were calculated by summing up each reviewer’s item scores within each domain and then standardizing them as a percentage of the maximum possible score according to the formula: (actual score - minimal possible score)/(maximal possible score - minimal possible score) × 100% (33). We performed a descriptive statistics analysis using the calculation of the total score by each reviewer and the score per domain. The data for each AGREE domain were given as medians and interquartile range (IQR). We defined the domain scores >80% as good scores, 60%~80% as sufficient scores, and <60% as low scores (34).

We calculated the inter-rater consistency of each domain using the two-way mixed intraclass correlation (ICC). According to the ICC results, the inter-rater consistency of each domain was divided into five grades, which were slight (0.01~ 0.20), fair (0.21-0.40), moderate (0.41-0.60), substantial (0.61-0.80), and very good (0.81-1.00) (35). All analyses were performed using SPSS version 26.0. A value of *p*<0.05 indicated a significant difference.

## Results

### Guideline characteristics

**Figure 1:**
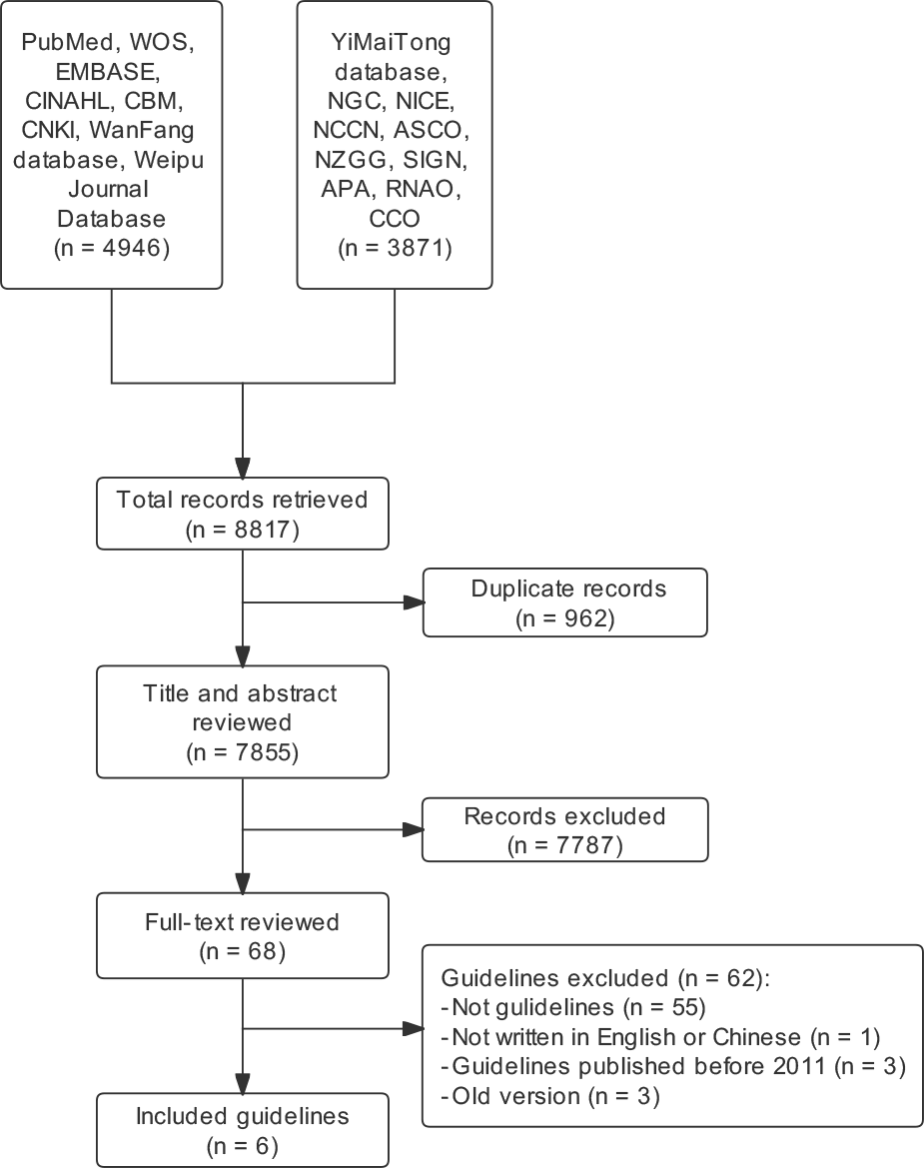
Guidelines selection process.

A total of 4964 records were yielded initially. Finally, six guidelines were included for evaluation after screening them by title, abstract, and full text (**Figure 1**). The years of publication for the guidelines spanned from 2011 to 2020. Guidelines were published from 2011 to 2020 by the following agencies: ASCO, European Palliative Care Research Collaborative (EPCRC), Canadian Association of Psychosocial Oncology (CAPO), National Comprehensive Cancer Network (NCCN), the Chinese Anti-Cancer Association’s Psychosocial Oncology Committee, and CCO. These guidelines focused on depressive symptoms in oncology patients, most of which also focused on anxiety symptoms. Three guidelines, CAPO, NCCN, and CPOS, covered psychological distress other than anxiety and depression. Half of the guidelines chose to use the GRADE system to evaluate the level of evidence (**Table 1**). The topic of the ASCO guideline (25) was about depression and anxiety. The EPCRC guideline (26) assisted palliative care professionals in managing the prevention, detection, diagnosis, assessment and treatment of depression in cancer patients. The CAPO guideline (27) focused on screening, assessing and dealing with distress and depression in adults with cancer. The NCCN guideline (28) provided the treatment guidance and follow-up directions for psychosocial distress in cancer patients. The CPOS (29) guideline mainly covered the clinical intervention of psycho-oncological issues, including coping with psychosocial stress, doctor-patient communication, referral for patients with psychosocial distress, hospice and palliative care, and so on. The CCO guideline (30) focus on integrating practical management tools to assist clinicians in delivering appropriate treatments for depression in patients with cancer.

### Quality appraisal of guidelines

The domain-standardized scores for selected guidelines and overall recommendations are presented in **Table 3**.

**Table 3:** AGREE II domain score of included guidelines.

Guideline ID	Scope and purpose	Stakeholder involvement	Rigour of development	Clarity and presentation	Applicability	Editorial independence	Overall assessment
ASCO, 2014 (25)	64%	85%	51%	99%	69%	94%	R
EPCRC, 2011 (26)	74%	65%	59%	67%	30%	98%	RM
CAPO, 2015 (27)	81%	65%	89%	93%	71%	63%	R
NCCN, 2020 (28)	76%	71%	84%	92%	84%	92%	R
CPOS, 2020 (29)	69%	22%	29%	74%	34%	58%	RM
CCO, 2016 (30)	64%	56%	64%	90%	77%	85%	R
Median score	71.5%	65%	61.5%	91%	70%	88.5%	—
IQR	64%-77.25%	47.5%-74.5%	45.5%-85.25%	72.25%-94.5%	33%-78.75%	61.75%-95%	
ICC(mean ± SD)	0.52 ± 0.27	0.79 ± 0.04	0.90 ± 0.06	0.77 ± 0.08	0.84 ± 0.14	0.84 ± 0.05	

ASCO, American Society of Clinical Oncology; EPCRC, European Palliative Care Research Collaborative; CAPO, Canadian Association of Psychosocial Oncology; NCCN, National Comprehensive Cancer Network; CPOS, Chinese Psycho-social Oncology Society; CCO, Cancer Care Ontario; IQR, interquartile range ICC, intraclass correlation coefficient; SD, standard deviation.

The ASCO guideline (25) received good scores in the stakeholder involvement (85%), clarity of presentation (99%) and editorial independence (94%) domains, sufficient scores in scope and purpose (64%) and applicability (69%), and low scores in the rigour of development (51%).

The EPCRC guideline (26) received good scores in the editorial independence domain (98%), sufficient scores in scope and purpose (74%), stakeholder involvement (65%) and clarity of presentation (67%), but low scores in rigour of development (59%) and applicability (30%) domains.

The CAPO guideline (27) received good scores in scope and purpose (81%), rigor of development (89%) and clarity of presentation (93%), and sufficient score in the stakeholder involvement (65%), applicability (71%) and editorial independence (63%) domains.

The NCCN guideline (28) received good scores in the rigour of development (84%), clarity of presentation (92%), applicability (84%) and editorial independence (92%) domains, and sufficient scores in the scope and purpose (76%), and stakeholder involvement domains (71%).

The CPOS (29) guideline received a sufficient score in the scope and purpose (69%), and clarity of presentation domains (74%), but low scores in stakeholder involvement (22%), rigour of development (29%), applicability (34%) and editorial independence (58%) domains.

The CCO (30) guideline received good scores in the clarity of presentation (90%) and editorial independence (85%) domains, sufficient score in scope and purpose (64%), rigour of development (64%) and applicability (77%) domains, with low scores in the stakeholder involvement (56%) domain.

### Scope and purpose

The median score for the scope and purpose domain was 71% (IQR 64%-77.25%). Most guidelines clearly described their overall objectives, health questions, and target populations. The ASCO (25) and CCO (30) guidelines received the lowest score.

### Stakeholder involvement

The median score for the stakeholder involvement domain was 61% (IQR 47.5%-74.5%). Only the CCO (30) and CPOS (29) guidelines scored under 60% for this domain. The CPOS (29) guideline did not describe their members’ roles in the guideline development process. The CAPO (27) and CCO (30) guidelines did not consider the views and preferences of the target population.

### Rigour of development

The median score for the rigour of the development domain was 63% (IQR 45.5%-85.25%). Only the CAPO (27), NCCN (28), and CCO (30) guidelines scored >60% because they used systematic methods of searching for evidence, and for formulating recommendations; Only the CAPO (27) and NCCN (28) guidelines described their procedures for updating guidelines. The CPOS (29) guideline received the lowest score because it did not describe the methods for formulating the recommendations and the procedures for updating guidelines.

### Clarity of presentation

Most guidelines provided specific, unambiguous, and easily identifiable recommendations. The median score for the clarity of the presentation domain was 86% (IQR 72.25%-94.5%). Only the EPCRC (26) guideline scored <70%.

### Applicability

The median score for the applicability domain was 39.3% (IQR 33%-78.75%). The ASCO (25), CAPO (27), NCCN (28), and CCO (30) guidelines scored >60% because they described the facilitators and barriers of their applications and sufficiently considered the costs of applying their recommendations. The EPCRC (26) and CPOS (29) guidelines did not provide any tools or suggestions for putting the recommendations into practice.

### Editorial independence

Most guidelines were developed with editorial independence. The median score for the editorial independence domain was 82% (IQR 61.75%-95%); only the CPOS guideline (29) scored < 60%. Although the CPOS (29) guideline reported its funding source, it did not specify the possible influence of funding on the content.

### Overall assessment

Based on the six domain scores and overall appraisal, the ASCO (25), CAPO (27), NCCN (28), and CCO (30) were recommended, and the EPCRC (26) and CPOS (29) guidelines were recommended with modifications.

### Consistency

ICCs for the AGREE II appraisal conducted by the four experts. The ICC values for the psychological distress guidelines appraisal ranged from 0.75 to 0.91 (**Table 3**). The “scope and purpose” domain scored the lowest (0.52), and “rigour of development”, “applicability”, “editorial independence domains” domains all scored ≥ 0.8, which indicated the inter-rater consistency was ideal.

## Discussion

Clinical practice guidelines are important tools that help clinicians and patients make evidence-based decisions about healthcare (36). Developing clinical practice guidelines for psychological distress among cancer patients is complex but necessary (37). In this study, we focused on the psychological distress among cancer patients, included six clinical practice guidelines, and used the AGREE II instrument to appraise these clinical practice guidelines. Generally, the clinical practice guidelines that were included in this study were of high quality, yet obscure differences in several dimensions were identified among guidelines.

The rigor of the development domain focuses on the process of gathering and synthesizing evidence, as well as the methods used to formulate and update recommendations (38), which is also one of the most important domains of assessing guideline implementation. The criteria for evidence selection and the process for updating the guideline, should be clearly described. However, in the CPOS guideline (29), we did not find the detailed expression of how to formulate the recommendations and how to update the guideline, instead majority of the content focused on disease treatment for psychological distress. Thus, this was why the CPOS guideline (29) scored the lowest in the domain of rigour of development as compared to other guidelines (**Table 3**).

Stakeholder involvement includes expert members of various disciplines, guideline users, and target groups. This domain mainly reflects the extent to which the guideline represents the views and willingness of the target population (such as patients, the public, and clinicians) (38). In this study, the CPOS guideline (29) disclosed only the names of experts but did not disclose their professions, which might lead to its low score in this domain (**Table 3**). It is crucial to consider patients’ perspectives on health care, as it has shown to have improved patient satisfaction and adherence to treatment (23, 39). However, we found that CCO guideline (30) didn’t disclose the target population of the guideline, which might result in the low score of stakeholder involvement.

The EPCRC (26) and CPOS (29) guidelines scored low in the applicability domain (**Table 3**), suggesting that these guidelines’ developers lacked the detailed information for physicians in clinical practice. Besides, we found that the CPOS guideline’s (29) editorial independence domain score (58%) was considerably lower than the other five guidelines (63% ~ 98%). It is possible that CPOS guideline (29) is lack of detailed information of the conflicts with interest, and the influence of funders on the guideline contexts is obscure. We also found that most of the guidelines scored well in the domain of scope and purpose as well as clarity of presentation. It may be because the guideline development groups have a consistent understanding of these domains and it is easier to fulfill the requirements of these domains.

From our study, there were still some issues that could be addressed in future guideline development and implementation. Firstly, as NCCN (28) and CCO (30) recommended, healthcare institutions could establish a collaborative multidisciplinary department including the oncologist and caregivers (nurse, psychologist, and social worker) to focus on the psychological distress in cancer patients (28, 30). Secondly, adopting the willingness of patients’ perspectives, which could be obtained through formal interviews with patients, and the public, or by reviewing literature which can demonstrate the views of relevant patients; and healthcare workers should assess the socio-demographic and cultural background of cancer patients (28), offering the individual interventions and humanistic care for psychological distress. Finally, guidelines should be updated regularly, as the NCCN does, possibly by establishing a guideline standing committee, searching the latest literature regularly, and updating the guidelines accordingly.

In this study, the recommended guidelines were ASCO (25), CCO (30), CAPO (27), and NCCN (28) according to the overall assessment. Importantly, we identified that the guidelines achieved the consensus on how to manage cancer patients’ psychological distress. The recommendations included, i. emphasize the importance of communication, especially family and friends’ support, ii. adapt cognitive-behavioral therapy (CBT), or other novel psychological interventions, iii. if necceray, pharmacologic interventions can be adapted (25, 30). Some recommendations have been applied in clinical practice and brought some improvement for psychological distress (40, 41). Meanwhile, we also reviewed the previous studies focusing on the psychological distress of cancer patients. Madineh et al. demonstrated that music therapy could alleviate cancer patients’ anxiety and depression (42). Besides, exercise intervention had positive effect on improving fatigue in breast cancer patients (43), and CBT could reduce psychological distress of adolescents and young adults after cancer treatment (44, 45). These results were consistent with the recommendations from the above guidelines. Thus, it is necessary to promote the generalization of psychological interventions on the psychological distress in cancer patients.

There were several advantages in our study. The expert group was composed of professionals with diverse disciplinary backgrounds, ranging from methodologists to clinicians. All of them were familiar with psychological distress in cancer patients, and capable of using the AGREE II instrument. Thus, the results of guidelines evaluation would be more objective and convincing. Meanwhile, our study also had some limitations. First, the guidelines that were published only in Chinese or English were searched for and included, while guidelines that were published in other languages were excluded. Second, the retrieval time of this study was over 12 months old when the review was accomplished, which reduced the timeliness of the study. It is a limitation of our study. Yet our group decided not to retrieved the latest literatures. The reason was that inconsistent results might emerge after inviting the experts to assess the updated guidelines for another time. It might change the conclusion of the present study. Besides, using the same search strategy, we have also conducted a quick check for the most recent guidelines in guideline databases published from January 2020 to present. The search result indicated that there was no new relevant guideline published by other organizations. It means that we have included all relevant guidelines. Third, the AGREE II instrument can only appraise the methodological content of the guideline without assessing the potential impact on the prognosis of patients (46).

## Data availability statement

The original contributions presented in the study are included in the article/**Supplementary Material**. Further inquiries can be directed to the corresponding authors.

## Author contributions

JH, YQ, and RH designed the research. HJ, JZ, and RZ collected and analyzed the data. RH, HJ, and JZ wrote the manuscript. YQ revised the manuscript. All the authors read and approved the final manuscript.

## Funding

This work was supported by the National Natural Science Foundation of China (grant number 72074067), Key project of Humanities and Social Sciences Research, Hebei Province (grant number ZD201908), Hebei Key Research and Development Project (19277799D, 21377729D), Natural Science Foundation of Hebei Province (H2020206483) and Technical Innovative Youth Talents of Hebei Medical University (grant number TJSK202103).

## Conflict of interest

The authors declare that the research was conducted in the absence of any commercial or financial relationships that could be construed as a potential conflict of interest.

## Publisher’s note

All claims expressed in this article are solely those of the authors and do not necessarily represent those of their affiliated organizations, or those of the publisher, the editors and the reviewers. Any product that may be evaluated in this article, or claim that may be made by its manufacturer, is not guaranteed or endorsed by the publisher.
