# Supplementary material for: Quality assessment of clinical practice guidelines on psychological distress of cancer patients using the AGREE II instrument
Source: Front Oncol. 2022 Aug 9;12:942219. doi: 10.3389/fonc.2022.942219 (PMC9396033; doi:10.3389/fonc.2022.942219)
Supplement: Supplementary file 1 [file Table_1.docx]

|  | Search term |
| --- | --- |
| #1 | cancer [Mesh] |
| #2 | distress [Mesh] |
| #3 | depression [Mesh] |
| #4 | anxiety [Mesh] |
| #5 | ((cancer*[Title/Abstract]) OR (neoplasm*[Title/Abstract])) OR (tumor*[Title/Abstract]) |
| #6 | (distress [Title/Abstract]) OR ("Emotional Stress"[Title/ Abstract]) |
| #7 | (depression*[Title/Abstract]) OR ("depressive symptom"[Title/Abstract]) |
| #8 | anxiet*[Title/Abstract] |
| #9 | guideline*[Title/Abstract] |
| #10 | #6 OR #7OR #8 |
| #11 | #5 AND #9 AND #10 |

Table S1 Search strategy for PubMed.

Table S2 Search strategy for CBM.

|  | Search term |
| --- | --- |
| #1 | ("guideline"[Field: intelligence]) AND 2011-2020[Date] |
| #2 | ("neoplasm"[Field: intelligence] OR "cancer"[Field: intelligence]) AND 2011-2020[Date] |
| #3 | ("anxiety"[Field: intelligence]) AND 2011-2020[Date] |
| #4 | ("depression"[Field: intelligence]) AND 2011-2020[Date] |
| #5 | ("distress"[Field: intelligence] OR " Emotional Stress "[Field: intelligence]) AND 2011-2020[Date] |
| #6 | #3 AND #4 AND #5 |
| #7 | #1 AND #2 AND #6 |

| Guideline database | Website |
| --- | --- |
| Yimaitong Guidelines Network | guide.medlive.cn/guideline/list |
| NGC | https://www.guideline.gov/ |
| NICE | https://www.nice.org.uk/guidance |
| NCCN | https://www.nccn.org/professionals/physician_gls/f_guidelines.asp |
| ASCO | https://www.asco.org |
| NZGG | https://www.health.govt.nz |
| SIGN | https://www.sign.ac.uk |
| APA | https://www.apa.org |
| RNAO | https://rnao.ca |
| CCO | https://www.cancercareontario.ca |

Table S3 Websites of guideline databases.

*NGC, National Guideline Clearinghouse; NICE, NCCN, National Institute for Health and Clinical Excellence; ASCO, American Society of Clinical Oncology; NZGG New Zealand Guidelines Group; SIGN Scottish Intercollegiate Guidelines Network; APA American Psychological Association; RNAO Registered Nurses' Association of Ontario; CCO Cancer Care Ontario.*
